# Metagenomic analysis revealed the bioremediation mechanism of lead and cadmium contamination by modified biochar synergized with *Bacillus cereus* PSB-2 in phosphate mining wasteland

**DOI:** 10.3389/fmicb.2025.1529784

**Published:** 2025-02-18

**Authors:** Yuxin Zhang, Jun Peng, Ziwei Wang, Fang Zhou, Junxia Yu, Ruan Chi, Chunqiao Xiao

**Affiliations:** ^1^Key Laboratory of Novel Biomass-Based Environmental and Energy Materials in Petroleum and Chemical Industry, Engineering Research Center of Phosphorus Resources Development and Utilization of Ministry of Education, School of Environmental Ecology and Biological Engineering, Wuhan Institute of Technology, Wuhan, China; ^2^Hubei Three Gorges Laboratory, Yichang, China

**Keywords:** phosphate mining wasteland, heavy metals, modified biochar, phosphate solubilizing bacteria, bioremediation

## Abstract

**Introduction:**

Phosphate mining wasteland is contaminated with heavy metals, such as lead (Pb) and cadmium (Cd), which pose significant environmental risks. Ecological restoration of these lands is crucial, but limited research has focused on the remediation of heavy metal-contaminated soils using modified biochar and functional microorganisms.

**Methods:**

In this study, we investigated the bioremediation of phosphate mining wasteland soil using modified biochar in combination with the phosphate-solubilizing bacterium *Bacillus cereus*. The effects of this synergistic approach on soil nutrient content, heavy metal immobilization, and microbial community structure were assessed.

**Results and discussion:**

The results indicated that the available phosphate content in the soil increased by 59.32%. The content of extractable state Pb^2 +^ and Cd^2 +^ decreased by 65.06 and 71.26%, respectively. And the soil nutrient conditions were significantly improved. Synergistic remediation can significantly increase the diversity and abundance of soil microbial communities (*p* < 0.05). *Janibacter*, *Lysobacter*, *Ornithinimicrobium*, *Bacillus*, and *Salinimicrobium* were the main functional flora during soil remediation, with significant correlations for the promotion of Pb^2 +^ and Cd^2 +^ immobilization and the increase of available phosphate and organic matter. *ZitB*, *czcD*, *zntA*, and *cmtR* are the major heavy metal resistance genes and regulate metabolic pathways to make microbial community function more stable after soil remediation in phosphate mining wasteland.

## 1 Introduction

Mining is a critical activity in mineral processing, energy development, metallurgical engineering, etc., which is essential for the strategic development of humankind (Chen J. et al., 2020; Sonter et al., 2020). Extensive phosphate mining activities form a large amount of solid waste, leading to the formation of more and more phosphate mining wasteland (Guo et al., 2021). Phosphate mining wasteland not only wastes valuable soil resources but also poses serious environmental challenges (Sun et al., 2018; Yuan et al., 2019). Among the contaminants, cadmium and lead have attracted considerable attention due to their high concentrations, persistence, and complex remediation processes (Peng et al., 2021). Both cadmium and lead can accumulate in the human body through the food chain as a result of long-term rainwater erosion, leaching, and diffusion into the soil (Qu et al., 2024; Qiu et al., 2021). Lead is a highly toxic neurotoxin that can cause severe health issues, including cancer and anemia, upon entry into the body (Yu et al., 2021; Marshall et al., 2020). Excessive cadmium exposure leads to accumulation in the liver and kidneys, resulting in hepatic damage and renal insufficiency (Howard et al., 2024). Consequently, there is an urgent need for efficient and environmentally friendly technologies to address the dual challenges of lead and cadmium contamination in soil. Such solutions are essential to safeguard human health and promote sustainable land use practices.

Various remediation technologies, such as soil leaching and phytoremediation, have been employed to remove heavy metals from contaminated soils (Tu et al., 2020). However, these methods have notable drawbacks, including high costs, the potential for secondary pollution, and lengthy remediation times, which limit their applicability (Cheng et al., 2024; Deng et al., 2024). Therefore, identifying efficient, cost-effective, and environmentally friendly remediation strategies is essential for addressing heavy metal pollution in mining areas. In recent years, microbial remediation has emerged as a promising approach due to its environmental benefits and lower costs (Che et al., 2024). Phosphorus-solubilizing bacteria (PSB), one of the most widely utilized functional strains, play a crucial role in mitigating heavy metal contamination (Zhao et al., 2023). Furthermore, these bacteria are capable of transforming insoluble phosphates derived from phosphate mining by-products into forms that are readily accessible for biological uptake through the secretion of organic acids (Chen et al., 2023a; Chen et al., 2023b). However, high heavy metal concentrations, such as lead and cadmium, can severely affect microbial cells, causing cell death through mechanisms like membrane disruption and DNA damage (Chen et al., 2023b; Shao et al., 2019; Jiang et al., 2020). Furthermore, the severe pollution and poor nutrient conditions prevalent in phosphate mining wasteland can weaken the remediation capacity of PSB and hinder their long-term colonization (Qi et al., 2023). Consequently, when faced with high concentrations of toxic cadmium and lead contamination from phosphate mining wasteland sites, microbial survival and reproduction often depend on complementary protective technologies.

Biochar, a carbon-rich material produced from organic waste through pyrolysis under limited oxygen, is a promising soil amendment that enhances carbon sequestration and soil fertility (Sun et al., 2020; Zhang et al., 2021). Its extensive surface area and porous structure make it an ideal habitat for microorganisms, enhancing microbial colonization (Singh et al., 2022; Luo et al., 2022). Studies have demonstrated that the combined application of biochar and PSB can address nutrient deficiencies and improve remediation efficiency (Lin et al., 2023). For instance, Qi et al. (2021) showed that bacterial-carrying biochar effectively immobilized uranium and cadmium, improving soil properties and microbial activity. Similarly, Chen et al. (2023b) found that swine manure biochar helped PSB better manage heavy metal stress, leading to higher removal rates of lead and cadmium compared to PSB alone. Despite these benefits, separating and recycling biochar after remediation remains a challenge (Reguyal et al., 2017). Magnetisation may be a good solution, as magnetizing biochar with iron oxides may not only effectively overcome its drawbacks but also improve its ability to remove pollutants (Yi et al., 2019). For example, Wang et al. (2015) found that the maximum adsorption capacity of magnetic biochar for arsenic was 428.7 mg/kg, approximately twice that of Biochar. Duan et al. (2022) achieved the recovery (100%) of iron-based modified biochar from soil and a 5.4% removal of Pb using dry magnetic separation. Therefore, the combination of biochar and heavy-metal-resistant phosphate-solubilizing bacteria can not only significantly enhance the remediation efficiency of heavy metal pollution in phosphate mining wasteland but also facilitate the recycling of biochar, offering an innovative and effective strategy for the treatment of contaminated sites.

In this study, a novel biochar-based adsorbent, BC-1, was developed using corn cob biochar (BC-0) as the raw material by combining phosphate-solubilizing bacteria (PSB) with the loading of Fe3O4. A comprehensive assessment was conducted on the combined remediation effect of BC-1 and heavy-metal-resistant phosphate-solubilizing bacterium PSB-2 (*Bacillus cereus*) on lead- and cadmium-contaminated soils in phosphate mining wasteland. The focus was on key parameters such as the immobilization of heavy metals, enhancement of soil nutrient profiles, and alterations in microbial community composition, gene expression, and functional potential. These aspects were further explored through metagenomic sequencing to provide a detailed understanding of the underlying microbial dynamics. The aim of this study was to provide a theoretical basis and technical support for the bioremediation of heavy metal pollution in phosphate mining wasteland.

## 2 Materials and methods

### 2.1 Soil, biochar, and PSB

The experimental soil was extracted from a phosphate mining wasteland in Yichang City, Hubei Province (111°1056′′–111°1217′ E, 31°1730′′- 31°20 00′′ N). It was air-dried, ground, and passed through a 2-mm sieve. The soil was then stabilized in a ventilated dry place for two weeks to assess its physicochemical properties for subsequent experiments. Soil physicochemical properties were determined after stabilization and the results are shown in Table 1. The corn cob biochar was prepared by holding at 500°C for 2.5 h under oxygen-limited conditions (Sha et al., 2023). The strain PSB-2, which was isolated and screened, has good phosphate solubilizing capacity as well as heavy metal Pb^2+^ and Cd^2+^ tolerance (Supplementary Figure 1). The strain had high homology (100%) with *Bacillus cereus*, with the accession number CP050183.1. PSB-2 was inoculated in sterilized LB medium (tryptic protein 10.0 g/L, sodium chloride 10.0 g/L, yeast infusion powder 5.0 g/L, and pH 7.0), activated and cultured to logarithmic growth stage (Chen and Achal, 2019).

**TABLE 1:** Physicochemical properties of soil and biochar before and after modification.

Property	Soil	BC-0	BC-1
pH	7.79 ± 0.02	8.69 ± 0.26	9.03 ± 0.14
Carbon content (%)	–	55.23 ± 1.37	39.54 ± 1.24
Fe (%)	–	0.12 ± 0.02	22.78 ± 0.76
Specific surface area (m^2^/g)	–	9.25 ± 0.65	54.32 ± 1.27
Total phosphate (g/kg)	24.70 ± 0.59	2.38 ± 0.13	4.95 ± 0.46
Available phosphate (mg/kg)	220.19 ± 19.62	17.41 ± 0.17	8.79 ± 0.39
Available Pb (g/kg)	110.97 ± 3.96	0.06 ± 0.01	0.03 ± 0.01
Available Cd (g/kg)	34.58 ± 0.21	0.03 ± 0.01	0.02 ± 0.01
Organic matter (g/kg)	22.13 ± 2.36	48.63 ± 2.41	39.29 ± 1.86
Cation exchange capacity (c mol/kg)	9.17 ± 0.06	35.65 ± 2.68	58.65 ± 1.63

“BC-0” refers to pretreated unmodified biochar, and “BC-1” refers to the composite modified biochar loaded with Fe_3_O_4_ after microbiological modification.

### 2.2 Preparation and characterization of modified biochar

After grinding and sieving, the corn cob biochar was washed with 0.1 mol/L HNO_3_ solution to remove ash from the pore structure. It was washed again with deionized water, then filtered and dried to get pretreated unmodified biochar (BC-0). Pretreated corn cob biochar BC-0 was incorporated into *Bacillus cereus* PSB-2 liquid LB medium cultured to logarithmic phase (biochar: medium = 1 g: 5 mL). It was then incubated at 30°C and 180 *r*/min for 48–72 h, filtered, washed, dried and ground. Microbiologically modified corn cob biochar was compositely modified by co-precipitation method loaded with Fe_3_O_4_ (Wang et al., 2021). Finally, it was filtered and washed with deionization, dried and milled to produce the composite modified biochar BC-1.

The surface morphology of BC-0 and BC-1 was examined using a field emission scanning electron microscope (Gemini SEM 300, Zeiss, Germany). Functional group characterization of the biochar before and after modification was performed using a Fourier-transform infrared spectrometer (NICOLET 6700, Thermo Fisher, USA). Additionally, lattice characteristics were analyzed using an X-ray diffractometer (D8 ADVANCE, Bruker, Germany).

### 2.3 Exploration of Pb^2+^ and Cd^2+^ adsorption in solution

Take 50 mL of mixed solution containing 200 mg/L Pb^2+^ and 50 mg/L Cd^2+^, add 0.10 g of the modified biochar BC-1 to the mixed solution, and oscillate at room temperature for 24 h to reach adsorption equilibrium. Then, the adsorbent that completed the adsorption was recovered with filter separation method, dried and ground. BC-1 before and after adsorption was subjected to X-ray photoelectron spectroscopy (XPS; ESCALAB XI+, Thermo Fisher Scientific, USA) to study the changes in elemental and bonding energies before and after adsorption on the adsorbent, as well as the adsorption mechanism. Besides, the recovered adsorbent particles were subjected to adsorbent desorption with 0.05 mol/L HCl solution and separated by filtration. The desorbed BC-1 was washed with deionized water, dried, ground and sieved. The adsorption-desorption operation was repeated for five cycles. The sorbent after five cycles of adsorption was then magnetically performed with a vibrating sample magnetometer (VSM; 8604, Lake Shore, USA) to analyze its magnetic stability.

### 2.4 Soil remediation experiments

Stabilized contaminated soil was divided into 11 cm pots with 500 g of soil per portion, and the concentration of adsorbent material or PSB-2 added to the soil uniformly was set at 2%, which was determined based on previous studies and further confirmed through preliminary experiments (Lahori et al., 2024; Li et al., 2023). Different treatments were set up, control (CK), PSB-2 (M), unmodified biochar (BC-0), modified biochar (BC-1), and PSB-2/modified biochar composite (MBC-1), with three parallel replications for each treatment. The soil remediation experiments were conducted by placing the soil in a naturally ventilated area at room temperature. After microbial colonization, the soil was replenished with distilled water every 5 days, maintaining the soil at 40% humidity. And 15 g soil sample was taken periodically from each experimental pot, dried, ground, and passed through a sieve for physicochemical determination. Continuous soil culture experiments were conducted for 55 days.

### 2.5 Determination of soil properties and heavy metal concentrations

The pH, total phosphate (TP), available phosphate (AP), extractable state Pb^2+^ and Cd^2+^ as well as soil organic matter (OM) and soil cation exchange capacity (CEC) of the soil samples were determined during the remediation process. The pH was measured using a pH meter (Zhang et al., 2020). TP was measured by ultraviolet spectrophotometry (UV-3600, Shimadzu, Japan) after digestion of soil samples with concentrated sulfuric acid and potassium persulfate, respectively (Afzal et al., 2020). AP was determined using ammonium vanadium molybdate colorimetric method after extraction with NaHCO_3_ (0.5 mol/L, pH 8.5) solution (Teng et al., 2019). Extractable state Pb^2+^ and Cd^2+^ were extracted by diethylenetriaminepentaacetic acid (DTPA) method (Tu et al., 2020). Then, they were determined using a flame atomic absorption spectrophotometer (ICE-3500, Thermo Fisher Scientific, Massachusetts, USA). OM and CEC were also determined for the remediated soil (Qi et al., 2021).

### 2.6 Soil macro-genomics analysis

After the completion of soil remediation at the phosphate mining wasteland, 10 g of fresh soil samples from each of the CK, M and MBC-1 were taken to extract microbial whole DNA from the soil using a soil DNA kit. Compared and analyzed in the cloud platform of Shanghai Major-bio Technology Co. The NR database and the Kyoto Encyclopedia of Genes and Genomes (KEGG) database were used for species, functional and genetic correlation analysis (Buchfink et al., 2015).

### 2.7 Data analysis

Phylogenetic analysis of the PSB-2 strain was conducted using the MEGA11 software to construct phylogenetic relationships. X-ray photoelectron spectroscopy (XPS) data were calibrated and peak fitting was performed using Avantage software (Thermo Scientific™). X-ray diffraction (XRD) data were analyzed with JADE 6.0. Each experimental set was repeated three times, and data were expressed as mean values with standard deviations. One-way ANOVA, utilizing Tukey’s method, was employed to assess the significance of differences in the data, using SPSS 26.0 for statistical analysis. All experimental data were visualized using Origin software.

## 3 Results and discussion

### 3.1 Physicochemical properties and characterization of modified biochar

The physicochemical properties of corn cob biochar before and after modification are shown in Table 1. After microbial modification and Fe_3_O_4_ loading modification, the pH of BC-1 was slightly increased to 9.03. The significant reduction of elemental C content and available phosphate content in biochar may be attributed to the growth and metabolism of phosphate solubilizing microorganisms, which played the role of microbial modification and released part of the available phosphate in the biochar (Chen et al., 2019). The Fe content in the modified biochar was significantly higher, accounting for 22.78%, indicating successful loading of Fe_3_O_4_. The larger the specific surface area, the better the adsorption effect of biochar (Zhao et al., 2020). The specific surface area of modified BC-1 was increased from 9.25 to 54.32 m^2^/g, which is a 5.87-times increase in specific surface area. Biochar has high organic matter content and cation exchange before and after modification, and can act as a soil conditioner, which is important for improving soil nutrient conditions.

**FIGURE 1:**
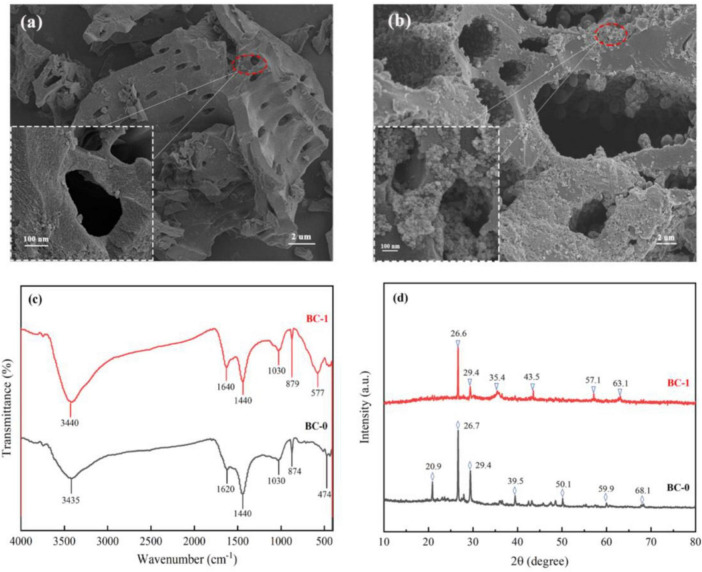
Characterization results of biochar before and after modification. SEM results of BC-0 **(A)** and BC-1 **(B)**. **(C)** FTIR results. **(D)** XRD results.

The scanning electron microscopy (SEM) results, illustrated in Figures 1A, B, unveil the intricate porous structure of biochar. This distinctive morphology not only provides convenient channels for the efficient transport of substances but also serves as a protective microenvironment that safeguards the reproductive processes of microorganisms, as previously highlighted by Quilliam et al. (2013). Moreover, Jiang et al. (2014) demonstrated that the high porosity of biochar enables robust interactions with metal ions through functional groups such as carbonyl, carboxyl, and hydroxyl groups, thereby enhancing its adsorptive capacity. Following microbial and loading modifications, a significant accumulation of fine particles was observed on the surface and within the pore structure of BC-1. This finding confirms the successful incorporation of Fe3O4 nanoparticles onto the modified biochar, which induces a notably rougher surface texture compared to its unmodified counterpart. This enhanced surface roughness significantly increases the number of available adsorption sites, thereby markedly improving the immobilization efficiency of Pb (II) and Cd (II) ions.

The functional group structure of biochar is closely related to its adsorption capacity, and FTIR was always used to qualitatively identify the characteristic functional groups of biochar materials (Yang et al., 2021). As shown in Figure 1C, the characteristic peak at 3,440 cm^–1^ corresponds to the -OH functional group, and the characteristic peak at 1,640 cm^–1^ corresponds to the C = O (Xiao et al., 2019; Sha et al., 2023). Other characteristic peaks near 1,440, 1,030 and 880 cm^–1^ correspond to C = C, C-O and C-H, respectively (Dai et al., 2020; Qi et al., 2021). The change in peak area after biochar modification proves that the increase in the type and number of functional groups can provide sufficient adsorption sites for heavy metal pollutants. A new characteristic peak appeared in BC-1 at 577 cm^–1^, which is attributed to the Fe-O and it is characteristic of Fe_3_O_4_ (Dong et al., 2018; Omer et al., 2023). In the XRD analysis results (Figure 1D), the diffraction peaks of BC-1 at 2θ = 35.4° (311), 43.5° (400), 57.1° (511) and 63.1° (440) are also attributed to Fe_3_O_4_ (Omer et al., 2023; Wang et al., 2016). The characterization results are all sufficient evidence of successful Fe_3_O_4_ loading in composite modified biochar.

### 3.2 Solution adsorption probing

**FIGURE 2:**
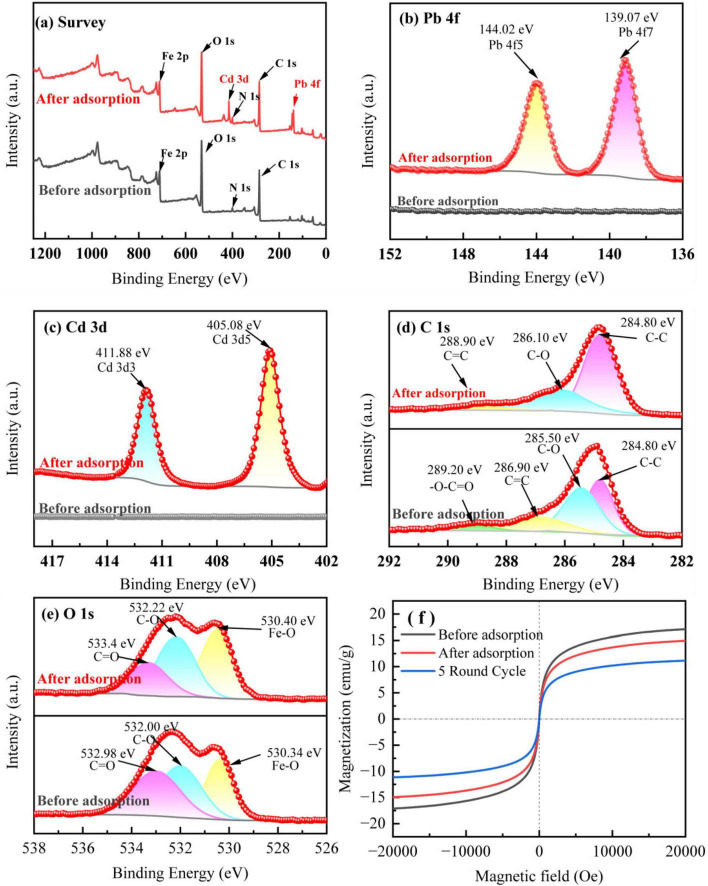
XPS results and hysteresis return lines before and after adsorption of BC-1. **(A)** Survey of the sample. **(B)** Pb 4f. **(C)** Cd 3d. **(D)** C 1s. **(E)** O 1s. **(F)** Hysteresis return line.

To further investigate the adsorption effect and mechanism of BC-1 on Pb^2+^ and Cd^2+^, the changes in the binding energy of BC-1 before and after adsorption were analyzed with XPS, and the magnetic stability of BC-1 before and after adsorption were investigated with VSM (Figure 2). In the full spectrum (Figure 2A), the major elements of modified biochar BC-1 before adsorption were C, N, O and Fe. After the adsorption experiments in solution, there were obviously more elemental absorption peaks of Pb and Cd in the spectrum, which proved that the modified biochar successfully adsorbed the heavy metals Pb^2+^ and Cd^2+^ in solution. As shown in Figures 2B, C, after adsorption, the binding energy of BC-1 at 139.07 eV corresponds to the Pb 4f7 orbital and at 144.02 eV is attributed to the Pb 4f5 orbital, respectively. The binding energies at 405.08 eV and 411.18 eV are attributed to the Cd 3d5 and Cd 3d3 orbitals, respectively.

In the C1s spectrum (Figure 2D), the peaks appearing at 284.80, 285.50, 286.90, and 289.20 eV in the BC-1 sample before adsorption represent C-C, C-O, C = C, and -O-C = O bonds, respectively (Huong et al., 2017; Gao et al., 2019). After adsorption, the binding energies of C-O and C = O appeared at 286.10 eV and 288.90 eV, respectively. After adsorption and immobilization of heavy metal ions, the relative peak areas of C-O and C = O decreased significantly and the binding energy of the corresponding bond increases, suggesting that the complexation of -OH and -COOH in the adsorbent plays an important role in adsorption (Sha et al., 2023). Besides that, -O-C = O disappeared from the peak area after the adsorption was completed, proving that π–π interaction also plays an important role in the adsorption of heavy metal ions (Liu et al., 2021). In the O1s spectrum (Figure 2E), the binding energies appearing at 530.34, 532.00, and 532.98 eV in BC-1 before adsorption are attributed to Fe-O, C-O, and C = O, respectively (Omer et al., 2023; Ji et al., 2022). After adsorption is complete, the binding energies corresponding to these bonds appear at 530.40, 532.22 and 533.40 eV. The change in the peak area and the shift in the binding energy of the bonds also proved the presence of complexation and ion exchange of oxygen-containing functional groups during the immobilization of heavy metal ions (Zheng et al., 2021).

The magnetic stability of BC-1 was evaluated by fitting the hysteresis return lines of BC-1 before and after adsorption, and the results are illustrated in Figure 2F. The saturated magnetization strength (Ms) of BC-1 was 17.05 emu/g before adsorption, and after one round of adsorption equilibrium, its saturated magnetization strength decreased to 14.89 emu/g. The results of the five cycles of adsorption testing demonstrated that the saturated magnetization strength of BC-1 remained at 11.10 emu/g, indicating that BC-1 exhibited excellent magnetic stability. Based on which, the magnetic adsorbent can be efficiently recovered in subsequent soil experiments in the presence of an applied magnetic field.

### 3.3 Soil remediation experiments

**FIGURE 3:**
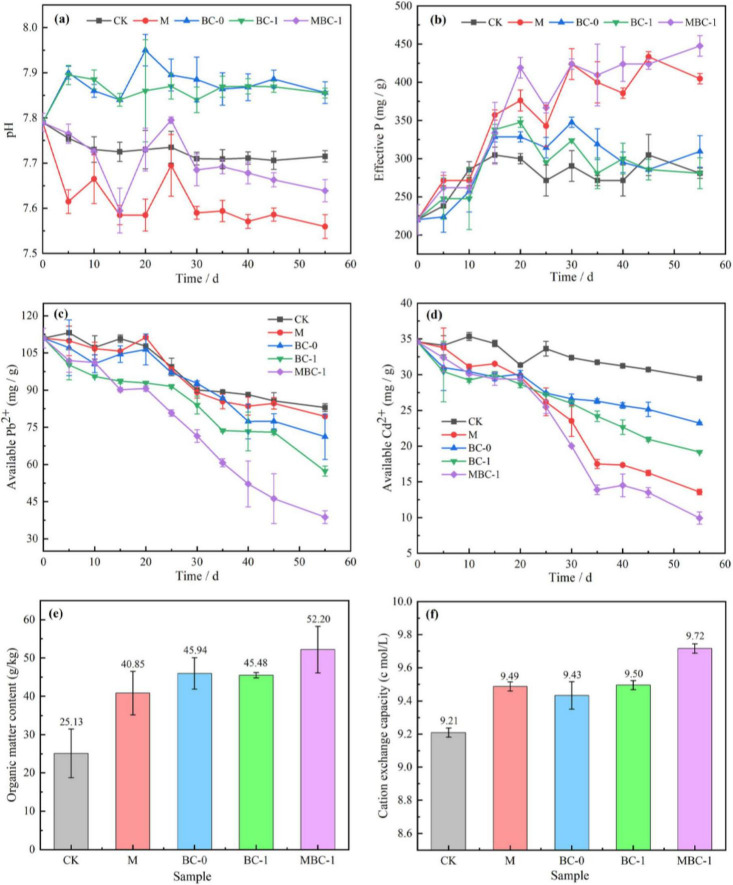
Physicochemical characterization during soil remediation. **(A)** pH change. **(B)** Available phosphate. Extractable state Pb^2+^ **(C)** and Cd^2+^ **(D)**. Soil organic matter content **(E)** and cation exchange capacity **(F)**.

The changes of physicochemical properties in soil remediation experiments are shown in Figure 3. From Figure 3A, the pH of BC-0 and BC-1 were increased compared to CK. It is due to the dissolution of alkaline substances in biochar into the soil, which regulated the soil pH (Tu et al., 2020). The soil pH decreased slightly in M and MBC–1, which is consistent with the study of Tu et al. (2020). Interestingly, the pH of the MBC–1 group dropped sharply on the 15th day. This could be attributed to the fact that after PSB–2 adapted to the new environment and stably colonized the soil, it produced a significant number of organic acids, resulting in changes in soil pH. This is consistent with the research of Xie et al. (2021). These organic acids, such as Gluconic acid, oxalic acid, malonic acid, citric acid and succinic acid, are common metabolites of PSB, which can dissolve insoluble phosphates, thereby reducing the pH value (Teng et al., 2019). Subsequently, on the 25th day, the pH value of the MBC–1 group increased rapidly, and then it decreased gradually. Yuan et al. (2011) reported a similar fluctuation in soil pH within the incubation with biochar from crop residues. They suggested the quick increase of soil pH was due to the dissolution of alkaline substances (such as inorganic carbonate) in the biochar, and then the pH was slightly changed after these readily released alkaline substances were depleted.

On the 55th day of the restoration, as shown in Figure 3B, the available phosphorus content in the soil of the M and MBC–1 groups reached 404.76 mg/kg and 447.62 mg/kg, respectively, an increase of 44.07 and 59.32% compared to the control group (CK). Numerous studies have demonstrated that secretion of organic acids by PSB is a critical microbial process that promotes phosphate solubilization (Gupta and Kumar, 2017). During bioremediation, the PSB-2 produces and releases small molecule organic acids by growing and metabolizing. The dissolution of insoluble phosphate in the soil into soluble phosphate promoted the release of phosphate. These findings are consistent with the results of the majority of previous studies (Lazo et al., 2017; Oteino et al., 2015). The fact that the available phosphorus content in the MBC–1 group is higher than that in the M group also indicates that biochar contributes to the increase in soluble phosphorus, which is consistent with the findings of Chen H. et al. (2020).

Changes in extractable state Pb^2+^ and Cd^2+^ content in soil remediation of phosphate mining wasteland are shown in Figures 3C, D. The contents of extractable state heavy metals Pb^2+^ and Cd^2+^ in MBC-1 were significantly reduced compared with CK. The concentrations of extractable state Pb^2+^ and Cd^2+^ in the soil of MBC-1 after remediation were 38.77 mg/kg and 9.94 mg/kg, which were reduced by 65.06 and 71.26%, respectively. The MBC-1 demonstrated the most effective remediation effect. It is proved that the synergistic remediation of modified biochar and PSB is an effective method to manage Pb and Cd pollution in soil.

The remediation of soil heavy metal contamination by modified biochar synergized with PSB involves a variety of mechanisms. Modified biochar can effectively reduce the mobility and bioavailability of heavy metal ions by physical adsorption, chemical complexation, ion exchange and precipitation (Gao et al., 2022). Its pore structure and elements (C, N, S, O, P, and Ca) provide habitat and nutrients for PSB growth (Beesley et al., 2011). PSB, in turn, can induce phosphate precipitation, and the released available phosphate directly immobilizes heavy metal ions in the soil, reducing their bioavailability (Ji et al., 2022; Li et al., 2021a). For example, Chen et al. (2016) isolated a strain of PSB *Bacillus cereus* 12-2 from lead and zinc smelting sites, which converted Pb into Ca_2.5_Pb_7.5_(OH)_2_(PO_4_)_6_ nanocrystals, confirming the biomineralization of Pb as hydroxyapatite. Besides, PSB can promote the release of available phosphate from biochar and dissolves both organic and inorganic phosphate by secreting enzymes and small-molecule organic acids (Li et al., 2018). The OH^–^, CO_3_^2–^ and PO_4_^3–^ ions produced during this process can form stable precipitates with heavy metal ions (Inyang et al., 2012). Phosphate precipitation, particularly metal-phosphate precipitation, is recognized as a key mechanism for heavy metal immobilization (Yang et al., 2021). However, PSB needs to be effectively protected in the complex soil environment to obtain the maximum effect of remediating heavy metal pollution. Biochar, by virtue of its large pores and strong adsorb ability, can reduce the loss of available phosphorus dissolved by phosphate solubilizing bacteria, which will help PSB to effectively fix Pb (II) and Cd (II) for a long time. Therefore, the synergistic effect of biochar with high phosphate content and PSB has a superior heavy metal stabilization ability in phosphate soil remediation, and the heavy metal elements will be immobilized in the biochar by forming complexes with phosphate (Álvarez-Rogel et al., 2018).

Soil organic matter content (OM) and soil cation exchange (CEC) are important indicators of soil fertility. Phosphate mining wasteland is impoverished and undernourished, making it difficult for other plants to survive. After the experiment, the OM as well as CEC was determined to assess the soil fertility improvement, and the results are shown in Figures 3E, F. The best soil fertility improvement was achieved in MBC-1, where soil nutrient conditions were greatly improved. Its soil organic matter content reached 52.20 g/kg, which was 107.72% higher than that of CK (25.13 g/kg), and the CEC was also significantly higher. Numerous studies have demonstrated that one of the most consistent responses after applying biochar (BC) is the increase in OM. This is mainly because biochar reduces the cycling rate of organic matter, or the organic matter is directly incorporated into the biochar (Lu et al., 2014; Qi et al., 2021). In addition, it has been revealed that the negatively charged functional group structure on the surface of biochar adsorbs cations, thus promoting an increase in soil cation exchange (Oliveira et al., 2017). Heavy metal cations in the phosphate mining wasteland soil are exchanged with cations in the biochar and immobilized as complexes in the biochar or precipitated in the soil, resulting in a decrease in the bioavailability of heavy metal ions (Jain et al., 2020; Qi et al., 2021). Therefore, the addition of biochar can also compensate for the soil infertility caused by heavy metal pollution, improve the soil microbial environment, and thus affect the stability of Pb and Cd in the soil.

### 3.4 Analysis of microbial communities

**FIGURE 4:**
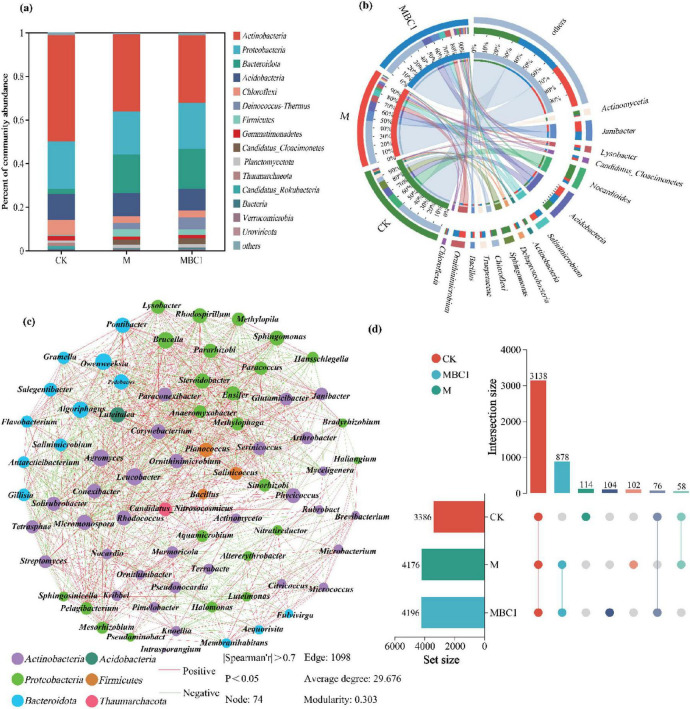
Analysis of soil microbial community. **(A)** Relative abundance analysis of microorganisms in different treatments (CK, M and MBC-1) (top 15 phyla). **(B)** Relative abundance analysis of microorganisms in different treatments (CK, M and MBC-1) (top 15 genera). **(C)** Microbial correlation network (genus level, abundance > 1%) and **(D)** Venn diagram analysis.

Alpha diversity of restored microbial communities was analyzed in the phosphate mining wasteland (Supplementary Table 1), while the microbial communities of M and MBC-1 changed considerably in comparison with CK. The diversity index (Shannon and Simpson), richness index (Chao 1) and evenness index (Pielou_e) gradually increased in CK, M and MBC-1, respectively. It was due to the fact that, not only *Bacillus cereus* was able to increase the diversity of microbial functions (Qin et al., 2015), but also biochar had a positive impact on microbial diversity (Xu et al., 2023). The increasing Pielou_e index reflected the increasingly even distribution of the community, while an excellent community coverage index (Coverage = 1) ensured the reliability of this sequencing result (Chen and Achal, 2019). The Venn diagram visualized the statistics of species unique or shared among the samples (Figure 4D). A total of 3,138 species were shared by CK, M and MBC-1, indicating that the microbial communities were extremely similar. More importantly, M and MBC-1 had extremely high similarity with 878 shared species. Moreover, the unique species of CK, M and MBC-1 were 114, 102, and 104, respectively, demonstrating the changes in microbial structure through different restorations.

Figure 4A showed the changes in the relative abundance of microbial phyla levels during the ecological restoration process in the phosphate mining wasteland. The results indicate that the M and MBC-1 groups significantly altered the composition of the microbial community. A total of 178 phyla were detected in soil samples. Among them, *Actinobacteria*, *Proteobacteria*, and *Acidobacteria* were the three phyla with the highest abundance in all samples. As studied in Wang et al. (2023), *Proteobacteria* and *Actinobacteria* were the dominant phylum in the Cd and Pb contaminated soils. Compared to CK, the relative abundances of *Bacteroidota*, *Deinococcus-Thermus*, *Firmicutes*, *Candidatus Cloacimonetes*, and *Planctomycetota* significantly increased in M and MBC-1, consistent with findings by Wu et al. (2019). Studies have shown that *Bacteroidota* can not only enhance the soil’s metal–fixing ability by regulating the physical and chemical properties of the soil (Li et al., 2024), but also increase the contents of available phosphorus and carbon in the soil through improving phosphorus dissolution and organic mineralization (Duan et al., 2020; Qin et al., 2020). Kruczynska et al. (2023) also pointed out that an increase in the abundance of *Bacteroidota* may indicate an improvement in soil quality. *Deinococcus-Thermus* is known to dominate the transformation of Cd fractions and regulate Pb mobility (Cui et al., 2021; Kavehei et al., 2022; Ren et al., 2021). *Firmicutes*, which contain a cluster of heavy metal tolerance genes, are commonly found in mining soils (Zhao et al., 2019). These phyla changes are closely linked to the application of biochar, which provides a favorable environment for microbial colonization, significantly influencing the abundance, diversity, composition, structure, and function of soil microorganisms (Zheng et al., 2022). Additionally, the MBC-1 group showed significantly higher numbers of *Bacteroidota* and *Deinococcus-Thermus* compared to CK and M, indicating that the combined remediation of PSB-2 and BC-1 not only enhanced the capacity to remediate cadmium and lead contamination but also improved soil quality.

Figure 4B presents the relative abundance of the top 15 microbial genera. Following remediation, the reduction of Pb and Cd concentrations led to a significant decrease in the relative abundances of sensitive and tolerant bacteria, such as Nocardia and *Sphingomonas*, which are key heavy metal-tolerant genera. In M and MBC-1, the relative abundances of Nocardia and *Sphingomonas* were significantly lower compared to CK. Conversely, the abundances of *Janibacter*, *Ornithinimicrobium*, *Salinimicrobium*, *Lysobacter*, and *Bacillus* increased in M and MBC-1. *Janibacter* (Vetrovsky and Baldrian, 2015) and *Lysobacter* (Hu et al., 2021) are particularly noteworthy, as they possess well-documented capabilities for remediating heavy metal-contaminated soils through various mechanisms, such as biosorption and biotransformation. The increase in these genera suggests a positive shift in the microbial community toward those that can actively contribute to soil decontamination efforts. Additionally, the elevated abundance of *Bacillus* is significant, as it indicates successful colonization by PSB-2.

Microbial network analysis showed the correlation between species (Figure 4C), which could obtain the coexistence of species in environmental samples and was significant for understanding the potential interactions between microorganisms within a community (Wood et al., 2017). Apparently, 74 nodes belonged to six different phyla, *Actinobacteria* (43.24%), *Proteobacteria* (32.42%), *Bacteroidota* (17.57%), *Firmicutes* (4.05%), *Thaumarchaeota* (1.35%), and *Acidobacteria* (1.35%). Furthermore, there were 1,098 edges, of which 53.73% were positive. *Bacillus*, which was added to the soil to participate in Pb and Cd remediation, showed significant positive correlation with *Planococcus*, *Salinicoccus*, *Leucobacter*, *Rhodococcus*, *Pelagibacterium*, and *Mesorhizobium*. *Bacillus* was already shown to be used in the remediation of heavy metal pollution with its ability to solubilize insoluble phosphate and excellent heavy metal resistance (Wani et al., 2019).

### 3.5 Correlation analysis of environmental factors

**FIGURE 5:**
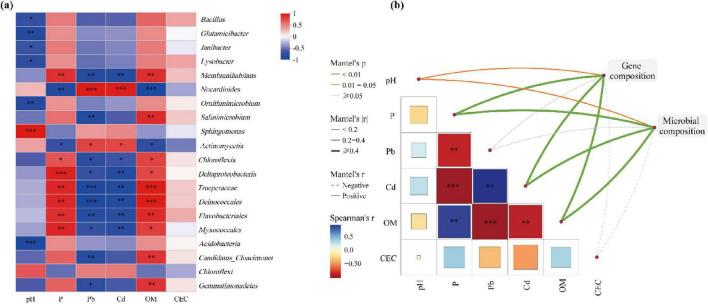
Correlation analysis between microorganisms and environmental factors. **(A)** Heatmap of correlations with environmental factors and microorganisms (top 20 genera). **(B)** Correlation of gene composition and microbial composition with environmental factors based on Mantel tests. Significance levels are denoted with *p < 0.1, **p < 0.01, ***p < 0.001.

According to the correlation analysis between microorganisms and environmental factors (Figure 5A), pH, AP, available Pb^2+^ and available Cd^2+^ showed high correlation with microorganisms. However, the correlation between CEC and microorganisms was low. As studied in Li et al. (2021b), OM, Cd, Pb, TP, and pH had significant effects on bacterial community composition and distribution. Soil pH was significantly positively correlated with *Sphingomonas* while negatively correlated with *Bacillus*, *Glutamicibacter*, *Janibacter*, *Lysobacter*, and *Ornithinimicrobium*. AP and OM showed significant positive correlation with *Membranihabitans*, but significant negative correlation with *Nocardioides*. Available Pb^2+^ and available Cd^2+^ had significant positive correlation with *Nocardioides* but significant negative correlation with *Membranihabitans*. Phosphate solubilizing microorganisms produced large amounts of organic acids, which led to the reduction of pH in the soil and the dissolution of insoluble phosphate, thus releasing soluble phosphate (da Silva et al., 2023).

There were correlations between environmental factors (Figure 5B). AP and OM significantly negatively correlated with available Pb^2+^ and available Cd^2+^; AP was significantly and positively correlated with OM; available Pb^2+^ and available Cd^2+^ showed significant positive correlation. These findings were highly consistent with the study of Jing et al. (2020). From the correlation of gene composition and microbial composition with environmental factors (Figure 5B), it was observed that the gene composition and microbial composition are significantly correlated with pH, AP, available Pb^2+^, available Cd^2+^, OM and CEC. OM affected the bacterial community structure and promoted the production of AP (Mohamed et al., 2022), which led to the precipitation of available heavy metal ions (Miretzky and Fernandez-Cirelli, 2008).

### 3.6 Species contribution to heavy metal resistance genes

**FIGURE 6:**
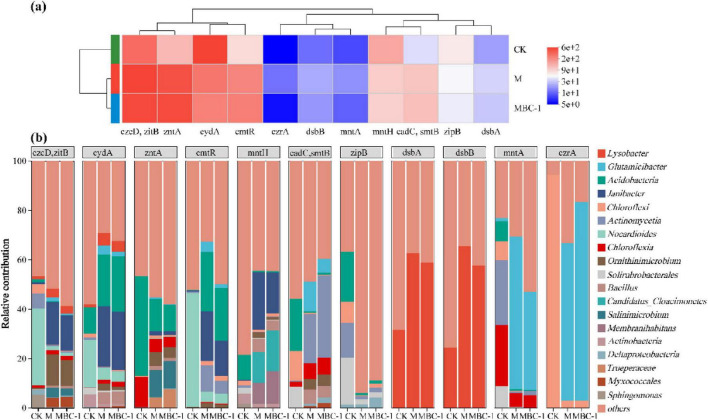
Analysis of heavy metal resistance genes in different treatments (CK, M and MBC-1). **(A)** Heatmap showing changes in the relative abundance of heavy metal resistance genes. **(B)** Species contribution of heavy metal resistance genes.

Figure 6 showed the relative abundance of different heavy metal resistance genes in different treatments as well as the species contribution. The relative abundance of *zitB*, *czcD*, *zntA*, *cmtR*, *cadC*, *smtB*, and *dsbA* in MBC1 was higher than that in CK compared to groups CK and M (Figure 6A). *zitB* and *czcD* transporters belong to the cation diffusion facilitator (CDF) family, which can transport Cd^2+^ (Chi et al., 2020). *zntA* is considered to be a Pb and Zn transporter protein ATPase with Cd and Pb resistance (Lee et al., 2001). *cmtR*, *cadC*, and *smtB* are not only transcriptional regulators of the ArsR family, but also Cd/Pb-responsive transcriptional repressors (Salam et al., 2020; Wang et al., 2005). *dsbA* is mainly involved in dithiol formation, and *dsbA* is mainly involved in dithiol formation, and *dsbA* is mainly involved in the formation of dithiols. *dsbA* is mainly involved in the formation of dithiols, which form thiol groups with high affinity for Cd (Stafford et al., 1999). The reduction of Cd and Pb concentrations in the MBC1 group was attributed to the high expression of these genes.

According to the species contribution of heavy metal resistance genes (Figure 6B), the species contribution of the same gene varied across treatments. *zitB*, *czcD*, *cydA*, *cmtR* in CK were the main contributors of *Nocardioides*. *Janibacter* was the main contributor of *zitB, czcD*, *cydA*, *cmtR*, *mntH* in M and MBC-1. *Janibacter* is the major contributor to *zitB*, *czcD, cydA*, *cmtR*, and *mntH* in M and MBC-1. *Lysobacter* is the major contributor of *dsbA* and *dsbB*. In M and MBC-1, *Corynebacterium glutamicum* was the major contributor of *mntA* and *czrA*. *Bacillus cereus* was involved in contributing *zitB*, *czcD*, *cydA*, *zntA*, *mntH*, *cadC*, *smtB*.

After remediation, the evolution of community structure also caused changes in the function of species, and microorganisms adapted to the changes in the growing environment by regulating various metabolic functions (Zhang et al., 2024). The function of the soil microbial community was predicted by PICRUSt (Supplementary Figure 2; Liu J. et al., 2018). Based on the secondary metabolic pathways, the relative abundance of amino acid metabolism, carbohydrate metabolism in treatments were higher, which were the foundational metabolic pathways necessary for microbial survival (Liu T. et al., 2018). Moreover, the exposure of heavy metals in soil caused an increase in amino acid metabolism (Qian et al., 2023). It has been demonstrated that most of the pathways of microorganisms would be reduced with increasing concentrations of heavy metals (Ma et al., 2022).

## 4 Conclusion

This study developed a novel biochar-based adsorbent, BC-1, from corn cob biochar (BC-0) through the synergistic combination of phosphate-solubilizing bacteria (PSB) and Fe_3_O_4_ loading. This modification significantly enhanced BC-1’s adsorption capacity for Pb^2 +^ and Cd^2 +^, while maintaining magnetic stability. Oxygen-containing functional groups (-OH, -COOH) and Fe-O bonds on the surface, along with π-π interactions, played a key role in ion exchange and complexation during adsorption. In combination with *Bacillus cereus*, BC-1 effectively remediated phosphate mining wasteland soil, increasing effective phosphate content (447.62 mg/kg) and reducing extractable Pb^2 +^ (65.06%) and Cd^2 +^ (71.26%). This treatment also improved soil organic matter and cation exchange capacity, enhancing soil health. Additionally, the remediation increased microbial community diversity and abundance, with *Janibacter*, *Lysobacter*, *Ornithinimicrobium*, *Bacillus*, and *Salinimicrobium* as the dominant groups. The upregulation of heavy metal resistance genes (*ZitB*, *czcD*, *zntA*, and *cmtR*) highlighted the microbial community’s robust response to Pb^2 +^ and Cd^2 +^ stress. Moreover, the stabilization of microbial function, especially in genetic processing, environmental processing, and metabolic pathways, supports the long-term efficacy of this remediation approach. This study suggests that combining PSB with modified biochar offers a promising, green, and sustainable solution for remediating heavy metal-contaminated soils in phosphate mining wastelands.

## Data Availability

The datasets presented in this study can be found in online repositories. The names of the repository/repositories and accession number(s) can be found in this article/Supplementary material.
