# Supplementary material for: Metagenomic analysis revealed the bioremediation mechanism of lead and cadmium contamination by modified biochar synergized with Bacillus cereus PSB-2 in phosphate mining wasteland
Source: Front Microbiol. 2025 Feb 18;16:1529784. doi: 10.3389/fmicb.2025.1529784 (PMC11876132; doi:10.3389/fmicb.2025.1529784)
Supplement: Supplementary file 1 [file Data_Sheet_1.docx]

***Supplementary Materials***

**Metagenomic analysis revealed the bioremediation mechanism of lead and cadmium contamination by modified biochar synergized with *Bacillus cereus* PSB-2 in phosphate mining wasteland**

Yuxin Zhang^1^, Jun Peng^1^**,** Ziwei Wang^1^**,** Fang Zhou^1^**,** Junxia Yu^1^**,** Ruan Chi^1, 2^**,** Chunqiao Xiao^1, 2*^

^1^Key Laboratory of Novel Biomass-Based Environmental and Energy Materials in Petroleum and Chemical Industry, Engineering Research Center of Phosphorus Resources Development and Utilization of Ministry of Education, School of Environmental Ecology and Biological Engineering, Wuhan Institute of Technology, Wuhan 430205, China

^2^Hubei Three Gorges Laboratory, Yichang 443007, China

*** Correspondence:**

Chunqiao Xiao

chunqiao@wit.edu.cn

**2. Materials and methods**

**2.1. Soil, biochar and PSB**

The experimental soil was extracted from a phosphate mining wasteland in Yichang City, Hubei Province (111^◦^1056′′-111^◦^1217' E, 31^◦^1730′′- 31^◦^20 00′′ N). The soil was naturally air-dried and grass roots, leaves as well as stones were removed. It was stabilized in a ventilated dry place for 2 weeks for subsequent experiments. Soil physicochemical properties were determined after stabilization and the results are shown in Table 1. The soil pH was 7.79 and the concentration of extractable state heavy metals Pb^2+^ and Cd^2+^ were 110.97 mg/kg, 34.58 mg/kg respectively with high Pb and Cd contamination. The corn cob biochar was prepared by holding at 500°C for 2.5 h under oxygen-limited conditions (Sha et al., 2023). The PSB was isolated and screened as PSB-2. The strain had high homology (100%) with *Bacillus cereus*, with the accession number CP050183.1. And the phylogenetic tree of the strain PSB-2 was constructed (Supplementary Figure 1(a)). The liquid environment was cultivated in an inorganic phosphate-selective medium with Ca_3_(PO_4_)_2_ as the sole source of phosphate. The strain PSB-2 has good phosphate solubilizing capacity as well as heavy metal Pb^2+^ and Cd^2+^ tolerance (Supplementary Figure 1). Luria-Bertani (LB) medium was configured by taking tryptic protein (10.0 g), sodium chloride (10.0 g), and yeast infusion powder (5.0 g) dissolved in distilled water (1000 mL, pH 7.0). PSB-2 was inoculated in sterilized LB medium, activated and cultured to logarithmic growth stage (Chen et al., 2019).


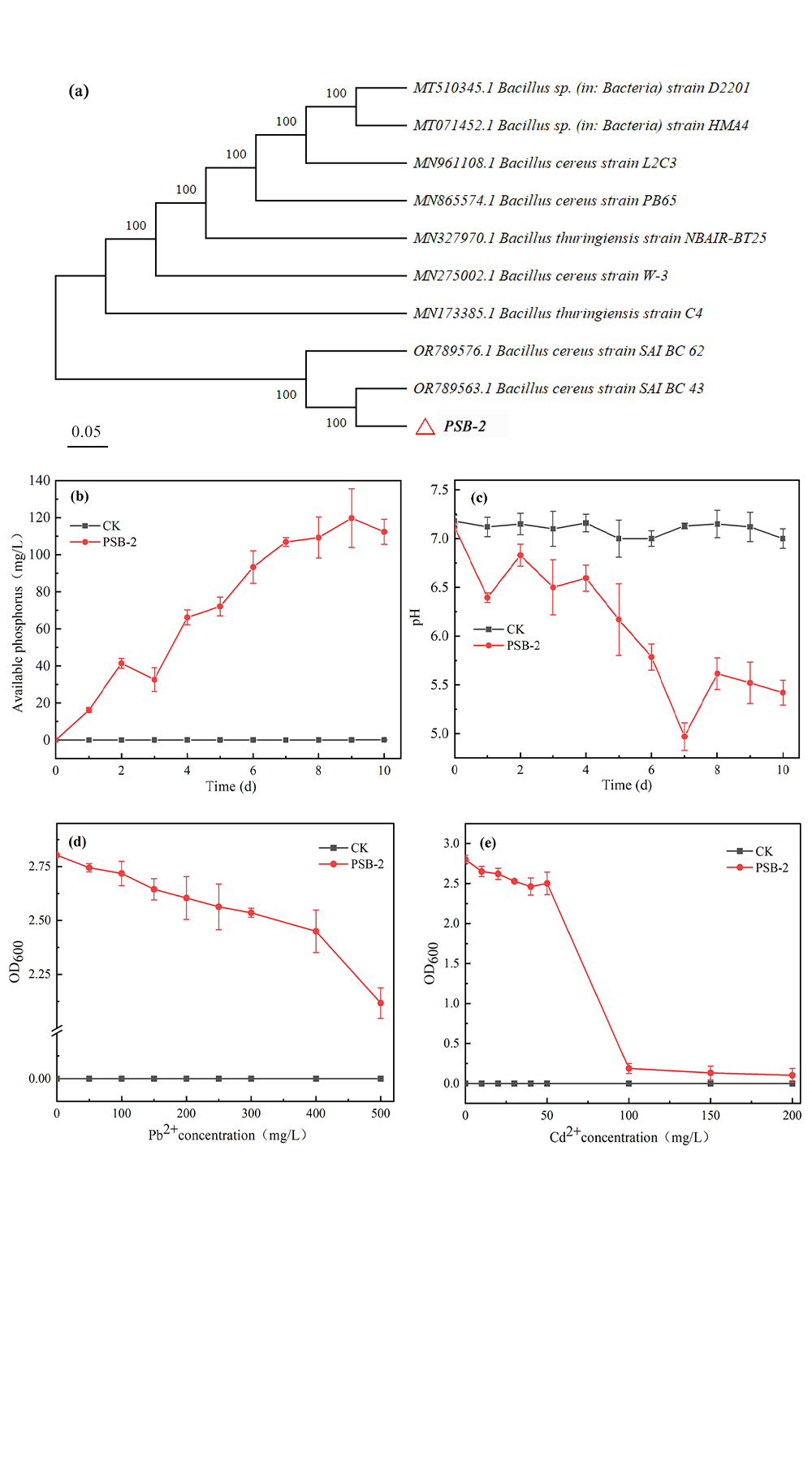


**Supplementary Figure 1**

Biochemical exploration of the phosphate solubilizing strain PSB-2. (a) Phylogenetic tree of strain PSB-2. Changes in available phosphate content (b) and pH (c) in the phosphate solubilization capacity experiment. Heavy metal Pb^2+^ (d) and Cd^2+^ (e) tolerance of the strain.

**3. 4. Analysis of microbial communities**

**Supplementary TABLE 1** Analysis of Alpha diversity index at genus level in different treatments. Results are presented as the mean ± standard deviation (n=3).

| **Sample** | **Shannon** | **Simpson** | **Chao 1** | **Pielou_e** | **Coverage** |
| --- | --- | --- | --- | --- | --- |
| CK | 4.12±0.04c | 0.0602±0.0028a | 3103.33±10.26b | 0.51±0.05c | 1 |
| M | 4.36±0.02b | 0.0431±0.0013b | 3865.67±24.34a | 0.53±0.01b | 1 |
| MBC-1 | 4.49±0.05a | 0.0363±0.0109c | 3870.67±17.01a | 0.54±0.01a | 1 |

**3. 6. Species contribution to heavy metal resistance genes**

After the remediation process of heavy metal pollution in phosphate mining wasteland, the evolution of community structure also caused changes in the function of species, and microorganisms adapted to the changes in the growing environment by regulating various metabolic functions (Zhang et al., 2024). The function of the soil microbial community was predicted by PICRUSt (Liu et al., 2018b) (Supplementary Figure 2). Primary metabolic pathways in CK, M and MBC-1 were dominated by metabolism (52.91%, 52.53% and 52.17%), genetic information processing (13.62%, 13.53% and 13.62%), environmental information processing (13.53%, 14.45% and 14.39%) and cellular processes (9.52%, 10.10% and 10.38%), respectively. Based on the secondary metabolic pathways, the relative abundance of amino acid metabolism (8.38%, 8.45% and 8.49%), carbohydrate metabolism (8.83%, 8.20% and 8.19%) in CK, M and MBC-1 were higher than others, which were the foundational metabolic pathways necessary for microbial survival (Liu et al., 2018a). Moreover, the exposure of heavy metals in soil caused an increase in amino acid metabolism (Qian et al., 2023). In contrast, the relative abundance of cell growth and death, carbohydrate metabolism, energy metabolism, xenobiotics biodegradation and metabolism, and metabolism of other amino acids were significantly reduced in relative abundance (*p* < 0.05), which indicated a more stable soil microbial community function and improved ecological stability. Otherwise, the relative abundance of other secondary metabolic pathways, such as Membrane transport, Replication and repair, etc., were significantly lower (*p* < 0.05) in CK, where available Pb^2+^ and available Cd^2+^ were higher. It has been demonstrated that most of the pathways of microorganisms would be reduced with increasing concentrations of heavy metals (Ma et al., 2022). Particularly, compared to M, the relative abundance of translation and biosynthesis of other secondary metabolites was significantly higher (*p* < 0.05) in MBC-1, which was boosted by the addition of biochar, while metabolism of cofactors and vitamins was significantly lower (*p* < 0.05).


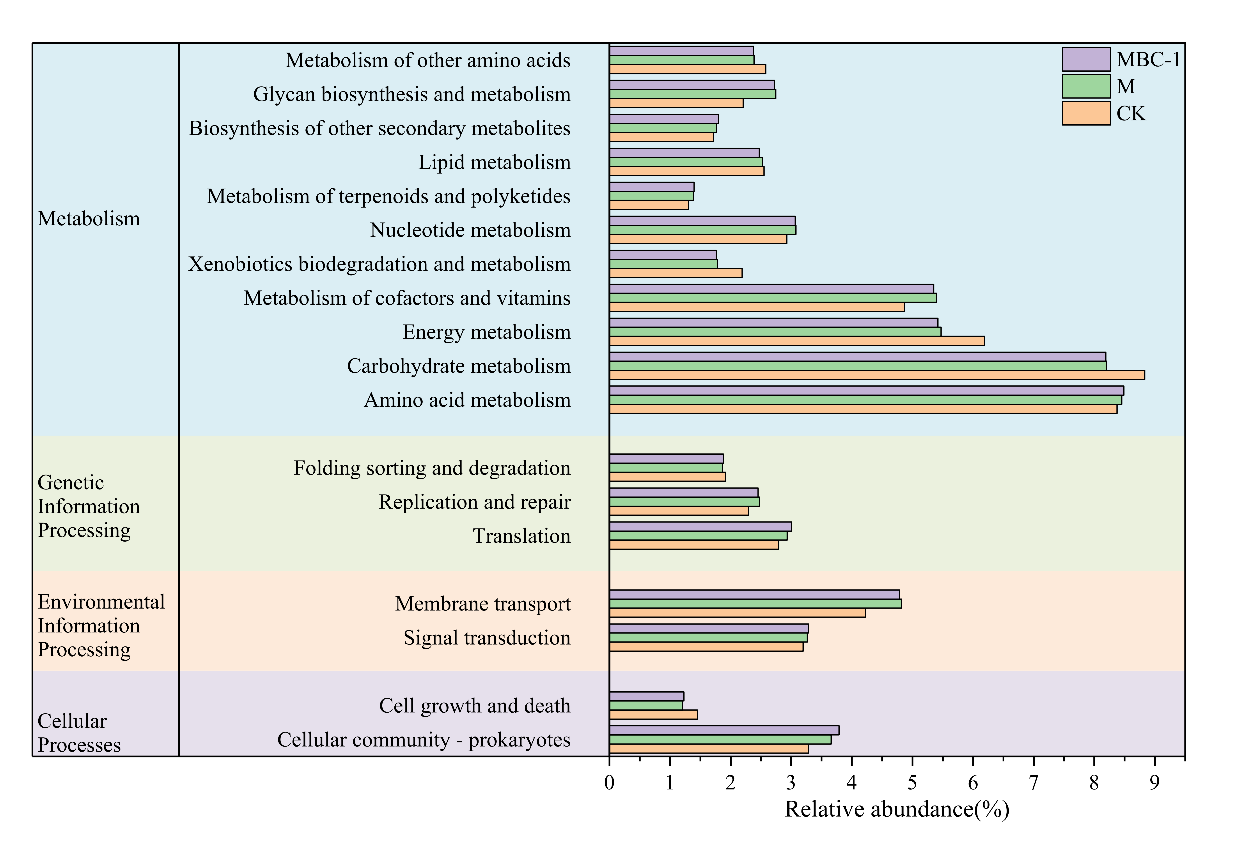


**Supplementary Figure 2**

Relative abundance (＞1%) of KEGG secondary metabolic pathways in soil microorganisms.
